# Childhood obesity prevention inequality: the dichotomy between health authorities’ provision and minority ethnicities’ perspectives

**DOI:** 10.1177/17579139251339088

**Published:** 2025-05-31

**Authors:** A Alkhatib, G Obita

**Affiliations:** Professor, College of Life Sciences, Birmingham City University, City South Campus, Edgbaston, Birmingham B15 3TN, UK; Hull-York Medical School, University of Hull

## Abstract

This topical paper covers important issue of health equity and obesity prevention and interventions in children from minority ethnicities. The authors highlight ongoing issues in this area to help health professionals and policy makers to improve population health.

## Main Discussion

The rising childhood obesity epidemic continues to be a global public health concern, especially in Western high-income countries (HICs).^1^ The UK has one of the highest rates of prevalence, and obesity in England among children aged 11−15 years, increased from 15% in 1995 to 24% in 2019.^2^ However, ethnicity disparity has paralleled such increase and has often been reported in health surveys across all age groups including children and adolescents in England.^3^ For example, it has been reported that compared with White children, Black African children were 40% more likely to be overweight, and Black and Asian children were 3 times more likely to have an obesogenic lifestyle than White children (odds ratio (OR) = 3.0, 95% confidence interval (CI) = 2.1−4.2 for Asian children; OR = 3.4, 95% CI = 2.7−4.3 for Black children), despite adjusting for deprivation and other sociodemographic characteristics.^4^ Children from an ethnic minority background have also been reported to have a higher prevalence of type 2 diabetes (T2D) than their other White ethnicity groups (1.42/100,000 vs 0.1/100,000).^5^ Recent systematic reviews have demonstrated significant disparities in childhood obesity comorbidities (hypertension, cardiovascular disease, and T2D), especially within HIC populations, which magnifies health inequalities in such developed healthcare systems.^6,7^ It is unlikely that such health disparities in obesity and its comorbidities can be resolved by existing health policies and prevention programmes.

**Figure:**
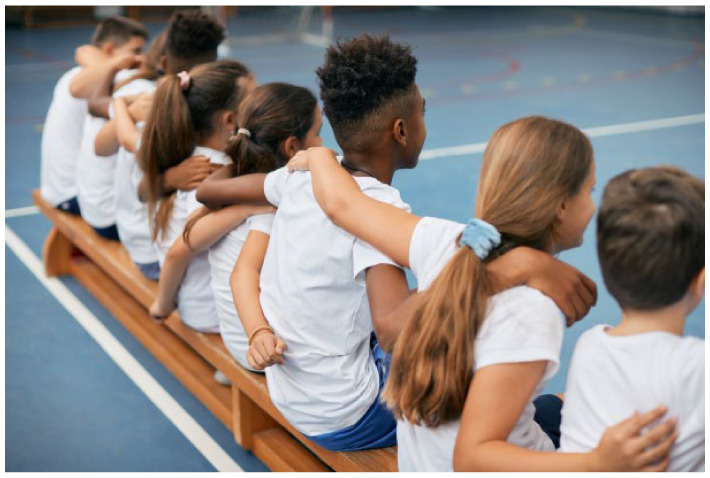


The UK government first recognised obesity as a public health concern in 1991, and several policies and strategies were since dedicated to address obesity. However, prevalence disparities in childhood obesity have continued to increase unabated despite significant funds and policy implementation (Table 1). Based on the prevalence rates alone, current evidence already demonstrates an inability of prevention strategies to address the childhood obesity burden, and associated health disparities. This is despite several recent attempts of prevention policies and intervention programmes, which have neither halted childhood obesity rise nor reduced disparities (Table 1).

**Table 1:** Examples of increased disparity in childhood obesity and its comorbidities despite NHS implemented policies and programmes

Childhood obesity policy and implementation period	Obesity prevalence among all children aged 10−11 years (source^8^)	Obesity prevalence among White vs. Black children aged 10−11 years during the same period (data source)^8^	Dichotomy of the implemented policy
Choosing a better diet – Choosing activity plan 2005−2008	17.5% (2006−2007) 18.3% (2008−2009)	19.4 % vs 26.7% (2006−2007) 17.3% vs 25.3% (2008−2009)	Poorly reported ethnicity gap, but 8% higher in Black than White ethnicities in 2006−2007 and 2008−2009
Healthy weight, Healthy life 2008–2010	18.0% (2008−2009) 19.0% (2010−2011)	17.3% vs 25.3% (2008−2009) 18.0% vs 26.7% (2010−2011)	Widened disparity: prevalence increased in Black (3%) more than White (1%) children
Healthy Lives, Healthy People − 2010–2015	19.0% (2010−2011) 20.0% (2015−2016)	18.0% vs 26.7% (2010−2011) 18.1% vs 28.9% (2015−2016)	Continuous widening of disparity (increased obesity among Black children by 2%, not White children)
Childhood Obesity: a plan for action – 2016−2018	20.0% (2015−2016) 24.0% (2018−2019)	18.0% vs 28.6% (2015−2016) 18.4% vs 28.9% (2018−2019)	Wider disparity: overall obesity increased by 4% but this was mainly among minority ethnicities, especially in Black ethnicities (increased by 7%)
Childhood Obesity: a plan for action, Chapter 2 – 2018−2019	24.0% (2018−2019) 26.0% (2020−2021)	18.4% vs 28.9% (2018−2019) 25.5% vs 35.7% (2020−2021)	Peak reached among ethnic minority children during pandemic
Childhood Obesity: a plan for action, Chapter 3 – 2019–current	23.0% (2022−2023)	21.4% vs 31.6% (2022−2023)	Reduction of obesity postpandemic but remains high among ethnic minority children

## Where is the Dichotomy?

1. *Clinical issues of intervention effectiveness*: This involves a weight-based system to address obesity, which fails to capture the secondary obesity outcomes such as risk factors of T2D, CVD, and hypertension. Obesity interventions are most effective when they add cardiometabolic comorbidities alongside weight as outcomes, especially when working with minority ethnicity high-risk populations.^7^ Interventions targeted towards minority ethnicity groups in the UK and elsewhere can follow established models of either early-phase intervention model, which targets large numbers based on prevalence on the prevalence of obesity risk factors, or late-phase intervention model which targets obesity comorbidities such as those with prediabetes and T2D.^9,10^
2. *Psychosocial barriers and lack of reach*: Obesity programmes, especially lifestyle nutrition or physical activity initiatives, have limited reach to minority ethnicities: this is due to the ivory tower approach used in allocating research funds to tackle obesity, lack of understanding and engagement with minority communities. This has led to almost giving up on the challenges of working with minority ethnicity communities due to the numerous cultural and psychosocial barriers.^11^ Conversely, minority communities feel they receive limited support from health authorities. Minority ethnicity parents of children with obesity comorbidities in the North East of England reported lack of reach and stigma as barriers to involvement in lifestyle interventions, despite their good knowledge and awareness of their children’s obesity status and what is needed to prevent associated comorbidities risks, including hypertension, fatty liver, and vascular disease.^12^ Contextualised obesity interventions that directly influence lifestyle behaviours among the disproportionately affected children from minority ethnic communities are urgently needed to reduce childhood obesity comorbidity risks.
3. *Primary care GP-centred limited advice*: Prevention programmes run by primary care centres and delivered through GP-centred limited advice are not effective. For example, non-specialised ‘healthy weight coaches’ were created to deliver ‘educational weight-based interventions’, and Complications from Excessive Weight (CEWs) clinics have proved ineffective in children from minority ethnicities.^13^ Obesity interventions, especially lifestyle interventions, should benefit from better engagement of specialists from non-traditional clinical fields of exercise physiologists, nutrition scientists, and well-trained allied health professionals.
4. *Disparity in research funding allocations to minority ethnicity researchers*: The current evidence from NIHR^14^ suggests researchers from an ethnic minority backgrounds are less successful in being awarded funding for research programmes, including obesity prevention programmes. Research leadership funding awards to ethnic minority groups is also behind, and with a lower representation in funding selection committees. Such funders’ lack of real engagement of minority ethnicity professional experts in decision-making hinders their potential contribution towards appropriating obesity intervention programmes, especially those aimed towards minority ethnicity populations. Wider engagement of underrepresented minorities and professions would certainly lead to better public health outcomes.

The current evidence on the impact of childhood obesity intervention programmes and policy implementation suggests a continuous widening health disparity among populations. There is a mismatch between health service obesity prevention approaches and the needs of high-risk minority ethnicity populations. Reducing such dichotomy contributes to reducing childhood obesity prevalence and wider health inequalities. Policies, programmes, and interventions need to be co-designed and co-produced by engaging ethnic minority populations, and multilevel involvement of stakeholders and cross-disciplines, especially from minority ethnicities. We have recently developed specific guidelines on childhood obesity in minority ethnicity populations, with an aim to inform public health policies, strategies, interventions, and implementation programmes.^15^ Those, alongside wider public health efforts, are essential to reduce health inequality and improve health-related outcomes.
